# Targeted Therapies and Immunotherapies for Diffuse Large B-Cell Lymphoma

**DOI:** 10.3390/cancers17152517

**Published:** 2025-07-30

**Authors:** Jahnavi Chaudhari, Nikesh N. Shah

**Affiliations:** 1 Department of Internal Medicine, HCA Florida Oak Hill Hospital, Brooksville, FL 34613, USA; jahnavi.chaudhari@hcahealthcare.com; 2 USF Health Morsani College of Medicine, Tampa, FL 33602, USA; 3 Department of Malignant Hematology, Tampa General Hospital Cancer Institute, Tampa, FL 33629, USA

**Keywords:** DLBCL, bispecific antibody, antibody–drug conjugate, immunotherapy, targeted therapy, glofitamab, polatuzumab vedotin, loncastuximab tesirine, relapsed/refractory DLBCL, relapsed/refractory, BCL2 inhibitors, molecular targeted therapy, B-cell lymphoma, treatment, molecular profiling

## Abstract

Diffuse large B-cell lymphoma (DLBCL) remains the most common subtype of non-Hodgkin lymphoma. While standard immunochemotherapy achieves high cure rates, up to 40% of patients experience relapsed or refractory (r/r) disease, highlighting the need for novel strategies. This review outlines emerging targeted therapies and immunotherapeutic approaches, excluding CAR-T. Bispecific antibodies have shown impressive efficacy, especially in combination with antibody–drug conjugates (ADCs), and are now being investigated in frontline DLBCL in combination with chemotherapy. ADCs like polatuzumab vedotin and loncastuximab tesirine have expanded treatment options in both first-line and salvage therapy. Combination therapy of small-molecule inhibitors—including BTK, BCL2, and EZH2 inhibitors—has efficacy in genomically defined subgroups. Collectively, these agents provide promising treatment options for patients with r/r DLBCL. Ongoing efforts are needed to elucidate long-term efficacy in newly diagnosed DLBCL, determine optimal sequencing of novel therapies in r/r DLBCL, and better expand access to bispecific antibodies across the globe.

## Simple Summary

Diffuse large B-cell lymphoma (DLBCL) is an aggressive lymphoma with a significant relapse rate despite standard chemoimmunotherapy. This manuscript reviews emerging targeted therapy and immunotherapies, particularly for patients who are unable to receive chimeric antigen receptor T-cell (CAR-T) therapy due to rapid disease growth, medical comorbidities, distance from a CAR-T center, or socioeconomic barriers. Bispecific antibodies and antibody–drug conjugates have shown promising efficacy, especially when used in combination together or with chemotherapy. Ongoing studies are further evaluating their use in newly diagnosed DLBCL.

## 1. Introduction

### 1.1. DLBCL Epidemiology and Subtypes

Diffuse large B-cell lymphoma (DLBCL) is the most common aggressive non-Hodgkin lymphoma (NHL). It comprises approximately 25% of all NHL cases [1]. In the United States, SEER data from 2018 to 2022 indicate that DLBCL had an age-adjusted incidence rate of 5.6 per 100,000 persons annually and a corresponding mortality rate of 1.7 per 100,000 [2]. Over 50% of cases were observed in individuals between 55 and 74 years of age [2]. The prognosis for patients with DLBCL can vary significantly, influenced by stage, International Prognostic Index (IPI) score, molecular subtype, and other comorbid conditions [1]. Efforts to subclassify DLBCL on the basis of cell of origin via immunohistochemistry (IHC) and high-grade B-cell lymphoma (HGBCL) by fluorescence in situ hybridization (FISH) have aided prognostication but are not comprehensive given the heterogeneity of these subgroups. Genomic subclassification of DLBCL by next-generation sequencing (NGS) appears to provide better prediction of therapeutic response; however, it is yet to be widely adopted in routine clinical practice [3].

### 1.2. Limitations of Standard Therapy and the Need for Novel Approaches in DLBCL

Despite initial responsiveness to chemoimmunotherapy, up to 30–40% of patients experience relapsed or refractory (R/R) disease [3]. Historically, salvage chemoimmunotherapy followed by High-Dose Therapy and Autologous Stem Cell Rescue (HDT/ASCR) was the treatment of choice for transplant-eligible R/R DLBCL patients [3]. However, patients with relapsed or refractory (R/R) DLBCL often have chemo-refractory disease or are not candidates for the rigor of transplant. Chimeric antigen receptor T-cells (CAR-T) have shown great efficacy with durable remissions in 30–40% of R/R DLBCL patients [4,5,6,7]; however, there are many real-world barriers to CAR-T therapy, including patient frailty or significant comorbidities, patient’s geographic proximity to a CAR-T center, cost of therapy, lack of caregiver support, and prolonged manufacturing times for those with rapid disease progression [8]. Additionally, adverse events (AEs) of CAR-T are also significant, including cytokine release syndrome (CRS), immune effector cell-associated neurotoxicity syndrome (ICANS), prolonged cytopenias, and infections. These challenges underscore the need for less toxic therapies that can be readily accessible and efficacious yet tolerable for older, frail patients [3]. Recently, bispecific antibodies and antibody–drug conjugates have shown promise in these regards [9]. In this article, we will review the evolving landscape of targeted therapies and emerging immunotherapeutic approaches (excluding CAR-T) in the management of DLBCL.

## 2. Bispecific Antibodies

**Figure 1:**
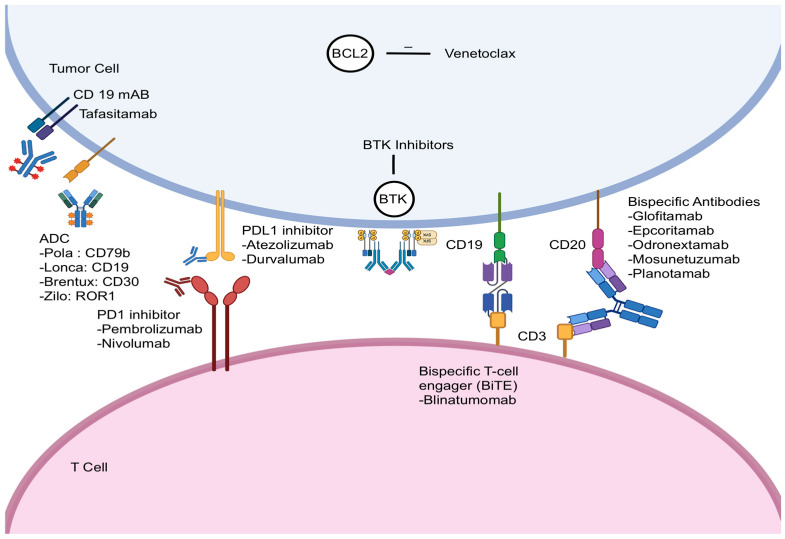
Therapeutic strategies in B-cell malignancies, illustrating various targeted therapies and immunotherapies. The diagram highlights agents such as BCL2 inhibitors (e.g., venetoclax), BTK inhibitors, antibody–drug conjugates (ADCs) targeting specific tumor antigens (e.g., CD79b, CD19, CD30, ROR1), immune checkpoint inhibitors that block PD-1 or PD-L1 pathways, CD19 monoclonal antibodies, and bispecific antibodies that bridge tumor cells (via CD19/CD20) with T cells (via CD3) to induce anti-tumor responses (Pola—polatuzumab vedotin; Lonca—loncastuximab tesirine; Brentux—brentuximab vedotin; Zilo—zilovertamab vedotin).

Bispecific antibodies (BsAbs) are engineered monoclonal antibodies designed to simultaneously bind two distinct antigens, enabling them to bridge different cell types or molecules. A structural configuration often features a single antibody fragment (Fab) containing both CD20- and CD3-binding sites arranged in a head-to-tail orientation, which enables them to engage with T-cells by binding to the CD3ε subunit of the T-cell receptor complex while simultaneously binding to the specific cell surface tumor antigen (CD20) on the target cancer cell (Figure 1). They induce T-cell activation through an MHC-independent mechanism without requiring antigen presentation via the MHC complex and T-cell receptor specificity. Once activated, these T-cells mediate cytotoxic responses against tumor cells through perforin and granzyme release [10]. Below is an overview of the most clinically advanced and approved bispecific antibodies, summarizing their pivotal trial data, efficacy outcomes, and safety profiles (Table 1 and Table 2).

### 2.1. Epcoritamab

Epcoritamab is a bispecific IgG1 monoclonal antibody designed to engage CD3 on T cells and CD20 on B cells, thereby promoting T-cell–mediated cytotoxicity against CD20-positive malignancies. In a phase I/II study of heavily pretreated relapsed/refractory (R/R) large B-cell lymphoma, single-agent epcoritamab achieved an overall response rate of ~63% with ~39% complete responses, demonstrating deep and durable remissions even in patients who had prior CAR T-cell therapy [11]. The treatment’s safety profile is manageable; the most common adverse effects are CRS (49.7%) (primarily grade 1–2, 2% grade 3+), injection site reaction, and neutropenia. ICANS is infrequent (seen in <10% of patients and typically low grade). This pivotal trial led to FDA approval in 3L+ DLCBL [11]. Epcoritamab is being investigated in various settings: in salvage therapy for transplant-eligible high-risk DLBCL, its combination with R-DHAX/C has produced high response rates (≥70% CR), and many patients proceeded to transplant [12,13]; in transplant-ineligible R/R DLBCL, epcoritamab combined with gemcitabine–oxaliplatin (GemOx) chemotherapy yielded an ORR of approximately 82% (with ~54% CR) in a phase II trial [14,15]. Promising results have also been seen in front-line therapy for patients unfit for standard R-CHOP: adding epcoritamab to an attenuated R-mini-CHOP regimen led to 89% overall response (82% CR) in an initial cohort of elderly/frail untreated DLBCL patients [16]. A recent study with the epcoritamab + R-ICE combination demonstrated high CR (65%) rates in pts with R/R DLBCL eligible for ASCT [17]. Epcoritamab and Pola-R-CHP combination in newly diagnosed DLBCL showed high ORR (100%) and CR (97%) in all subgroups of DLBCL with no high-grade CRS [19]. Novel chemo-free combinations are under exploration as well—for instance, fixed-duration epcoritamab plus lenalidomide has shown encouraging activity in R/R DLBCL [18]. Ongoing studies are broadening the scope of epcoritamab’s application, including a randomized phase III trial comparing epcoritamab to standard-of-care salvage regimens (investigator’s choice of rituximab–gemcitabine–oxaliplatin or bendamustine–rituximab) in R/R DLBCL [22].

### 2.2. Glofitamab

Glofitamab is a CD20 × CD3 T-cell engaging bispecific antibody designed with a novel 2:1 structural format, with two regions that bind to CD20 on B cells and one region that binds to CD3 on T cells. In the pivotal phase II study, glofitamab monotherapy in relapsed/refractory (R/R) DLBCL achieved an overall response rate (ORR) of 52% and complete response (CR) rate of 39%, with a manageable safety profile dominated by low-grade cytokine release syndrome (CRS). Extended follow-up demonstrated that responses were durable, with a median CR duration of 24.1 months and 38% of patients remaining in CR at 3 years [24,25,26]. These data led to FDA approval of glofitamab in 3L + DLBCL. Importantly, glofitamab has shown efficacy in patients who relapsed after CAR-T therapy, as demonstrated in a LYSA phase II trial [27]. In the phase III STARGLO study, glofitamab plus GemOx significantly improved median overall survival (25.5 vs. 12.9 months) and CR rates (58.5% vs. 25.3%) versus R-GemOx in transplant-ineligible R/R DLBCL with sustained response in updated 2-year outcomes [116]. Though approved by the European Medical Association and incorporated into NCCN guidelines in the second-line setting, the combination was not FDA-approved due to low US patient representation in the trial [23]. Glofitamab–polatuzumab vedotin combination showed a CR rate of 59% and sustained remissions in high-risk populations, including those with high-grade B-cell lymphoma and prior CAR-T exposure [28]. A phase Ib study of glofitamab + Pola-R-CHP in untreated DLBCL demonstrated promising efficacy and tolerability [29], and the ongoing SKYGLO phase III trial is assessing this combination in a randomized setting [30]. Similarly, the addition of glofitamab to R-CHOP in newly diagnosed high-risk DLBCL (defined by ctDNA dynamics) resulted in high response rates and a favorable safety profile in the phase I study [31]. In the transplant-eligible setting, glofitamab combined with R-ICE has shown efficacy and manageable toxicity, supporting its use in bridging or salvage strategies [32]. The COALITION trial and its randomized phase II follow-up showed that frontline glofitamab-containing regimens yielded deep and durable metabolic remissions [34,35]. Additionally, at EHA 2025, a separate phase II study evaluating the glofitamab–rituximab–polatuzumab combination in patients unfit for R-CHOP revealed high ORR with fewer adverse effects [117,118].

### 2.3. Odronextamab

Odronextamab is an off-the-shelf CD20 × CD3 bispecific antibody demonstrating promising efficacy in relapsed/refractory diffuse large B-cell lymphoma (DLBCL), including after CAR T-cell therapy. In the phase I ELM-1 study, single-agent odronextamab achieved an ORR of 49% and a CR rate of 31% in patients with DLBCL who progressed post-CAR T, with durable responses and manageable cytokine release syndrome (CRS) through step-up dosing [36]. In the phase II ELM-2 trial, odronextamab achieved an ORR of 52% and a CR rate of 31% in heavily pretreated R/R DLBCL patients (≥2 lines of therapy), with a median duration of response of 9.5 months [37]. Ongoing phase III trials are now evaluating odronextamab in earlier lines of therapy: OLYMPIA-3 is comparing odronextamab-CHOP to R-CHOP in newly diagnosed DLBCL [38], while OLYMPIA-4 is assessing odronextamab vs. standard-of-care regimens in R/R aggressive B-cell lymphoma [39].

### 2.4. Mosunetuzumab

Mosunetuzumab, a CD20 × CD3 bispecific antibody, has demonstrated significant therapeutic potential in diffuse large B-cell lymphoma (DLBCL) across various clinical contexts. As a single agent, it is both active and tolerable for patients with relapsed or refractory (R/R) disease [40] and has yielded promising, durable complete responses in elderly or unfit patients as a first-line treatment [42]. When used as monotherapy, the most common adverse events include cytokine release syndrome (CRS), occurring in approximately 26–29% of patients (predominantly low-grade and early in treatment), and grade 3–4 neutropenia, seen in about 10–13% of elderly/unfit patients. The utility of mosunetuzumab extends to combination therapies, where it has shown favorable safety and efficacy when paired with CHOP chemotherapy for previously untreated patients [41]. In a phase 1b/2 trial, Mosun-Pola achieved an ORR of 59% and a CR rate of 45.9%, with responses observed in patients previously treated with CAR-T therapy [38]. A randomized phase II study further highlighted its superiority over rituximab plus polatuzumab, with higher ORR (78% vs. 50%) and CR rates (58% vs. 35%) [43,44]. Common adverse events for this combination are generally manageable and include grade 3–4 neutropenia (20–30% of patients), fatigue (~35–43%), and low-grade CRS (10–18%) [43,44]. This promising regimen is being further evaluated in the phase III SUNMO trial, showing a median PFS of 11.5 months vs. 3.8 months with 25.9% CRS (<1% grade 3) [45]. The low rate of high-grade CRS with mosun/pola makes this combination appealing for outpatient administration, potentially facilitating future uptake in the community setting. The versatility of mosunetuzumab is underscored by ongoing clinical trials exploring novel combinations with agents such as loncastuximab tesirine [42] and a triplet regimen with polatuzumab vedotin and lenalidomide, reflecting its growing importance in the DLBCL treatment [41].

### 2.5. Planotamab

Plamotamab is another CD20 × CD3 bispecific antibody, which is currently under investigation for relapsed/refractory (R/R) B-cell non-Hodgkin lymphoma. In the phase I study, intravenous plamotamab achieved an ORR of 47% and a CR rate of 26% in a heavily pretreated population [49]. Cytokine release syndrome (CRS) occurred in 57% of patients, although 94% of events were grade 1–2 and only 3% experienced grade ≥ 3 CRS. Other common treatment-emergent adverse events included pyrexia (57%), chills (35%), and neutropenia (32%) [49]. A 2024 update demonstrated that subcutaneous administration was well tolerated, with no new safety signals and lower peak cytokine levels compared to intravenous dosing [50]. Plamotamab is now being evaluated in a randomized phase II trial in combination with tafasitamab and lenalidomide vs. tafasitamab–lenalidomide in R/R DLBCL [51].

### 2.6. Blinatumomab

Blinatumomab, a CD19 × CD3 bispecific T-cell engager, has demonstrated modest single-agent activity in DLBCL. In a phase II study of relapsed/refractory (R/R) DLBCL, blinatumomab achieved an ORR of 43% and a CR rate of 19%, but responses were typically short-lived (median duration: 2.9 months). Adverse events were frequent, with CRS occurring in 37% and neurologic events in 58% of patients, including 22% with grade ≥ 3 neurotoxicity [52]. To enhance efficacy and reduce relapses, combination strategies have been pursued. In a phase Ib trial, blinatumomab-pembrolizumab showed an ORR of 36%, with improved outcomes in PD-L1–positive tumors [53]. As consolidation post-autologous stem cell transplant, blinatumomab was associated with a 1-year progression-free survival of 72% and low-grade CRS and neurologic toxicity in <10% of patients [54]. In newly diagnosed high-risk DLBCL, blinatumomab post–R-CHOP demonstrated feasibility with manageable safety—grade ≥ 3 neurologic events occurred in ~10% [55]. Ongoing trials are also evaluating its combination with lenalidomide to enhance T-cell activation in R/R non-Hodgkin lymphoma [56]. The limited durability of response, toxicities, and logistics of continuous infusion have limited the clinical uptake of blinatumomab as monotherapy in DLBCL.

Despite the promising efficacy of bispecific antibodies, especially in combination with chemotherapy or ADCs, their widespread adoption has been limited due to multiple challenges, namely clinical and logistical barriers, particularly for standalone community practices. Inpatient admission for step-up dosing can be burdensome, especially for sites new to bispecific administration without an affiliated hospital. Inpatient and outpatient multidisciplinary teams and workflows are critical in early identification and management of CRS and ICANS but can be challenging to establish across multiple sites. Strategies to mitigate CRS and ICANS risks, such as subcutaneous administration, prephase steroids or obinutuzumab, and combination with chemotherapy or ADCs, appear to reduce risks and allow more patients to be treated in the outpatient setting. Infection risk is an ongoing challenge, especially for indefinite therapies. Ongoing efforts such as prophylactic immune globulin administration have proven to reduce infection risk in BCMA-directed bispecifics, but further investigation is needed in lymphoma [119].

Optimal patient selection and sequencing of therapies are rapidly evolving areas of investigation. With the plethora of clinical trials investigating bispecific antibody use in earlier lines of therapy, ideal combination strategies will be challenging to select without randomized trials. For patients treated with bispecific antibodies in early lines of therapy, it will be crucial to study the impact on subsequent therapies, namely CAR-T, as there are potential concerns regarding T-cell fitness and exhaustion. There are preliminary retrospective data from the French DESCAR-T registry suggesting similar response rates and survival for bispecific-treated and bispecific-naïve patients who went on to receive subsequent CAR-T, but further investigation is warranted [120]. Translational efforts will be critical to assess the role of molecular subtypes and tumor microenvironment on outcomes, ideally influencing optimal patient selection. For example, a multicenter retrospective cohort found dismal outcomes with epcoritamab and glofitamab in CD20-negative patients, with a median PFS of 1.3 months and a median OS of 2.0 months [121].

## 3. Checkpoint Inhibitors

### 3.1. Atezolizumab

Atezolizumab, a PD-L1 immune checkpoint inhibitor, has been explored in multiple settings in diffuse large B-cell lymphoma (DLBCL). In the consolidation setting, a phase II study evaluated atezolizumab in high-risk DLBCL patients in complete remission after R-CHOP, reporting a 2-year progression-free survival of 72.4% [57]. A phase Ib trial combining atezolizumab and obinutuzumab in R/R DLBCL showed an overall response rate (ORR) of 17%, with manageable toxicity [58]. Another phase Ib study of atezolizumab plus tazemetostat reported an ORR of 16%, with responses more frequent in EZH2-mutant tumors [59]. In the frontline setting, atezolizumab added to R-CHOP showed a CR rate of 77% and 2-year PFS of 70%, though with increased immune-related adverse events such as rash (27%) and transaminitis (18%) [60]. For transformed or Richter’s DLBCL, combinations with venetoclax and obinutuzumab (MOLTO trial) achieved ORRs of 67–74% [62,63]. While the efficacy of atezolizumab as monotherapy remains limited, its combination with other chemo/immunotherapy agents has shown promising results.

### 3.2. Durvalumab

Durvalumab, a PD-L1 checkpoint inhibitor, is being explored primarily as a combination regimen in diffuse large B-cell lymphoma (DLBCL). In a phase II study, durvalumab combined with R-CHOP or with lenalidomide plus R-CHOP was evaluated in previously untreated high-risk DLBCL patients, demonstrating acceptable safety and feasibility, though detailed efficacy (ORR 54–67%) data were limited [66]. Additionally, a phase I study assessed durvalumab with the CD19-targeted antibody–drug conjugate loncastuximab tesirine in relapsed/refractory B-cell lymphomas, including DLBCL, showing preliminary anti-tumor activity with manageable toxicity [67]. While early results are promising, durvalumab’s role in DLBCL remains investigational.

### 3.3. Pembrolizumab

Pembrolizumab is a PD-1 immune checkpoint inhibitor, which has revealed limited efficacy as monotherapy in relapsed/refractory (R/R) DLBCL with response rates around 20% [71]. However, it has shown greater efficacy in select subtypes; in the final analysis of KEYNOTE-170, pembrolizumab achieved an overall response rate (ORR) of 41% and a complete response (CR) rate of 20% in relapsed/refractory primary mediastinal large B-cell lymphoma (PMBCL), with durable responses and manageable toxicity [68]. In the PORTIA phase Ib study, pembrolizumab given with CAR T-cell therapy (tisagenlecleucel) improved the expansion and persistence of CAR T cells, with a 50% ORR and acceptable safety profile [70]. Combinations with BCR inhibitors (e.g., acalabrutinib) or HDAC inhibitors (e.g., vorinostat) in R/R DLBCL have also yielded ORRs ranging from 26% to 55%, with enhanced responses observed in patients with PD-L1 expression [73,74]. Moreover, pembrolizumab is being explored in frontline therapy; in a phase I trial combining it with R-CHOP, a 2-year progression-free survival of 83% and a CR rate of 77% were observed in treatment-naïve DLBCL [76]. It is also under investigation for patients relapsing after CAR T-cell therapy, where it may enhance immune reactivation [78].

### 3.4. Nivolumab

Nivolumab, a PD-1 immune checkpoint inhibitor, has shown modest activity in diffuse large B-cell lymphoma (DLBCL), particularly in select subsets and in combination regimens. In a phase II study of patients with relapsed/refractory DLBCL ineligible for or relapsed after autologous stem cell transplant, nivolumab monotherapy achieved an overall response rate (ORR) of 10%, with a median duration of response of 11 months, indicating limited single-agent efficacy [79]. However, it has shown greater efficacy in combination regimens. The BeGeRN regimen (nivolumab with bendamustine, gemcitabine, and rituximab) showed modest responses (ORR 45%) in heavily pretreated patients with R/R B-cell lymphoma [80]. In high-grade B-cell lymphoma with MYC and BCL2 and/or BCL6 rearrangements, nivolumab consolidation after DA-EPOCH-R induction was well tolerated and showed 61% CMR [81]. The CheckMate 436 trial showed that the nivolumab–brentuximab vedotin combination in relapsed/refractory primary mediastinal large B-cell lymphoma yielded an ORR of 70% and a CR rate of 43% [82].

## 4. Antibody–Drug Conjugates

### 4.1. Polatuzumab Vedotin

Polatuzumab vedotin (Pola), an antibody–drug conjugate targeting CD79b, has reshaped frontline therapy for diffuse large B-cell lymphoma (DLBCL) through the landmark POLARIX trial. This phase III study compared Pola-R-CHP (polatuzumab, rituximab, cyclophosphamide, doxorubicin, and prednisone) against standard of care R-CHOP in previously untreated intermediate- to high-risk DLBCL. At the 5-year follow-up, Pola-R-CHP demonstrated a sustained progression-free survival (PFS) benefit, with a 5-year PFS of 70.2% vs. 62.4% for R-CHOP (hazard ratio 0.73) [83,84]. Notably, in post hoc subgroup analyses, the magnitude of benefit was more pronounced in patients with higher IPI scores (3–5), double-expressor lymphoma, and the non-GCB subtype, though genomic clustering appears to better delineate the C5 cluster with a vastly superior response to Pola-R-CHP [83,84,122]. While overall survival (OS) remained similar between arms (5-year OS ~78% in both groups), the Pola-R-CHP arm significantly reduced the risk of relapse or progression with a comparable safety profile and delayed the need for subsequent therapies [83,84]. These durable results confirm that Pola-R-CHP offers a long-term disease control advantage and support its use as a new standard of care in frontline DLBCL. Beyond the frontline setting, polatuzumab has also shown efficacy in various combinations for relapsed/refractory DLBCL, including with bendamustine–rituximab [85], venetoclax [100], bispecific antibodies, and salvage chemotherapy regimens such as Pola-R-GemOx [87] and Pola-R-ICE [91,92]. The predominant additional toxicity appears to be neuropathy, mostly low grade.

### 4.2. Loncastuximab Tesirine

Loncastuximab tesirine (Lonca) is an antibody–drug conjugate targeting CD19, approved for relapsed/refractory (R/R) diffuse large B-cell lymphoma (DLBCL) after ≥ 2 prior therapies. In the pivotal phase II LOTIS-2 trial, Lonca demonstrated an ORR of 48% and a CR rate of 25%, with a median duration of response of 13.4 months [101]. This study led to its accelerated approval for R/R DLBCL in 2021. Combination strategies are under investigation to enhance efficacy: in the LOTIS-3 trial, Lonca + ibrutinib showed preliminary ORRs of 57% in R/R DLBCL patients, including those with prior CAR T-cell therapy [102]. Lonca is being evaluated in combination therapies as well, including Lonca + rituximab (Lonca-R) vs. R-GemOx [103], Lonca-Pola, and Lonca-Mosun [104]. The LORELY phase II study is evaluating Lonca in patients with progressive disease after CAR T-cell therapy, highlighting its potential as a salvage option in post-cell therapy failure settings [105]. The LOTIS-7 trial of lonca + glofitamab showed a promising ORR of 93.3% (86.7% CR) with 41% CRS (2.4% G3) and 7.3% low-grade ICANS, suggesting a potential new combination strategy for transplant-ineligible patients unable to receive CAR-T [123].

### 4.3. Brentuximab Vedotin

Brentuximab vedotin (BV), an antibody–drug conjugate targeting CD30, has shown clinical activity in diffuse large B-cell lymphoma (DLBCL), particularly in CD30-expressing tumors. In a phase II study, BV monotherapy in relapsed/refractory DLBCL achieved a modest response (ORR 44%, CR 17%) [106]. However, in the ECHELON-3 trial, brentuximab vedotin (BV) combined with lenalidomide and rituximab achieved an overall response rate (ORR) of 64% and a complete response (CR) rate of 40% in heavily pretreated relapsed/refractory DLBCL patients [107]. Common adverse events included neutropenia (43%), fatigue (36%), and diarrhea (30%); grade ≥ 3 events occurred in 67%, with peripheral neuropathy in 18%—mostly low grade [107]. Further studies of BV in combination with R-CHP are ongoing [108,109].

### 4.4. Zilovertamab Vedotin

Zilovertamab vedotin (ZV) is an antibody–drug conjugate targeting ROR1, which is being evaluated in diffuse large B-cell lymphoma (DLBCL). In the waveLINE-004 phase II study, ZV monotherapy in relapsed/refractory DLBCL achieved a modest response (ORR 30%, CR 10%) with manageable toxicity; the most common adverse events were neutropenia, fatigue, and peripheral neuropathy [114]. Ongoing trials are exploring ZV in combination with R-CHP in both frontline (waveLINE-010) [111] and GCB-subtype-specific [110] settings, as well as in relapsed/refractory disease in combination with R-GemOx (waveLINE-003) [113]. These studies aim to establish ZV as a novel ROR1-directed therapy across DLBCL treatment lines.

### 4.5. Naratuximab Emtansine

Naratuximab emtansine is an antibody–drug conjugate against CD37, a lymphocyte surface marker expressed in B-NHL, linked to a cytotoxic maytansinoid, DM1. In the phase 2 trial, the combination of naratuximab plus rituximab showed modest efficacy of 44% with a CR of 24% in patients with R/R DLBCL who were not candidates for transplant and failed prior lines of therapy [115]. However, no further clinical trials are going on at this point in time.

**Table 2:** Efficacy and safety outcomes of select novel therapies in relapsed/refractory diffuse large B-cell lymphoma (DLBCL).

Trial/Regimen	Indication/Line	N (Active Arm)	ORR (%)	CR (%)	Median PFS (Months)	Median OS (Months)	CRS (Any Grade, %)	CRS (Grade 3+, %)	ICANS (Any Grade, %)	ICANS (Grade 3+, %)
**Epcoritamab Monotherapy** (EPCORE NHL-1) [11]	3L+ R/R DLBCL/LBCL	157	63.1	39.5	4.2 (overall); 37.3 (CR pts)	18.5 (overall); NR (CR pts)	51	2.5–3	6–6.4	0.6 (1 fatal event)
**Epcoritamab + GemOx** (EPCORE NHL-2) [15]	2L+ ASCT-ineligible R/R DLBCL	103	85	61	11.2 (overall); 26.7 (CR pts)	21.6 (overall); NR (CR pts)	52	1	2.9	1 (G3)
**Glofitamab Monotherapy** (NP30179) [24,25,26]	3L+ R/R DLBCL/LBCL	155	59	38	1-yr PFS in CR: 71% PFS—CR at EOT 80%	NR (18-mo OS: 41%; 1-yr OS in CR: 92%)	64	4	8	3
**Glofitamab + GemOx** (STARGLO) [23]	2L+ R/R DLBCL (ASCT-ineligible)	183 (Glofit-GemOx arm)	68.3	58.5	13.8	25·5 (18·3–NE)	44.8	2.3	2.3	0.6
**Mosunetuzumab + Polatuzumab Vedotin** (Phase 1b/2, NCT03671018) [43,44]	2L+ R/R LBCL	98 (Phase 2 expansion)	59.2	45.9	11.4	23.3	16.7	3.1	1 (G2 seizure)	Not reported
**Mosunetuzumab + Polatuzumab Vedotin** (SUNMO) [45,46]	2L+ R/R aNHL (DLBCL, HGBCL, trFL, FL3B)	138 (NCT05171647)	70.3	51.4	11.5 months	23.2 months	25.9	0.7	0	0
**Polatuzumab Vedotin + Bendamustine + Rituximab** (Pola-BR, GO29365) [85]	3L+ R/R DLBCL (ASCT-ineligible)	40	62.5	52.5	9.2 month	12.4	N/A	N/A	N/A	N/A
**Polatuzumab Vedotin + R-GemOx** (POLARGO) [87]	R/R DLBCL (ASCT-ineligible)	Not specified	52.7	40.3	7.4	19.5	N/A	N/A	N/A	N/A
**Loncastuximab Tesirine Monotherapy** (LOTIS-2) [101]	3L+ R/R DLBCL/LBCL	145	48.3	24.8	4.9(1-yr EFS 54%)	9.5 (1-yr OS 74%)	N/A	N/A	N/A	N/A
**Brentuximab Vedotin + Lenalidomide + Rituximab** (ECHELON-3) [107]	3L+ R/R DLBCL (ASCT/CAR-T ineligible)	155	70.9	45.6	5.8	15.9	N/A	N/A	N/A	N/A
**ViPOR** (Venetoclax + Ibrutinib + Prednisone + Obinutuzumab + Lenalidomide) [124]	R/R DLBCL	50	54	38	Not reported (2-yr PFS 34%)	Not reported (2-yr OS 36%)	N/A	N/A	N/A	N/A

Abbreviations: R/R = relapsed/refractory; DLBCL = diffuse large B-cell lymphoma; LBCL = large B-cell lymphoma; ASCT = autologous stem cell transplant; CR = complete response; ORR = overall response rate; PFS = progression-free survival; OS = overall survival; CRS = cytokine release syndrome; ICANS = immune effector cell-associated neurotoxicity syndrome; NR = not reached; NE = not estimable; N/A = not available.

## 5. Monoclonal Antibody

### Tafasitamab

Tafasitamab is an Fc-modified, humanized monoclonal antibody (mAb) directed against the pan B-cell antigen CD19 [125]. The L-MIND trial was an important trial which led to FDA approval of tafasitamab lenalidomide combination in ASCT-ineligible patients (pts) with R/R DLBCL [126]. It showed overall ORR was 57.5% (n = 46/80), including complete response (CR) in 40% of pts. Long-term data revealed durable response with manageable safety profile. Other ongoing trials include tafasitamab + lenalidomide combination in patients with relapsed/refractory DLBCL (firmMIND) [127], tafasitamab + lenalidomide + R-CHOP vs. R-CHOP for newly diagnosed DLBCL (frontMIND) [128].

## 6. BTK Inhibitors

Bruton’s tyrosine kinase (BTK) is an enzyme crucial for the activation of the B-cell receptor signaling pathway. BTK inhibitors function by interfering with this pathway, thereby preventing the uncontrolled proliferation of abnormal B cells and inducing their death. In the PHOENIX trial, ibrutinib in combination with R-CHOP was evaluated for newly diagnosed non-GCB DLBCL. This study initially did not meet its primary endpoint, showing no improvement in event-free survival (EFS) in the overall intent-to-treat (ITT) or activated B-cell (ABC) DLBCL populations. However, in patients younger than 60 years, ibrutinib plus R-CHOP improved EFS, PFS, and OS with slightly higher adverse events. In patients aged 60 years or older, ibrutinib plus R-CHOP was associated with increased toxicity, leading to compromised R-CHOP administration and worse outcomes [129]. More specifically, in younger patients with the MCD and N1 genetic subtypes of non-GCB DLBCL, the addition of ibrutinib to R-CHOP resulted in remarkable 100% three-year EFS and OS rates, a substantial improvement compared to R-CHOP alone (MCD: 48% EFS, 69.6% OS; N1: 50% EFS and OS) [130]. This highlights that the initial trial results, while seemingly negative, masked a clear benefit for a specific patient population, emphasizing the critical role of molecular subtyping in guiding therapy. Acalabrutinib has also shown efficacy in de novo ABC DLBCL patients in a phase 1 trial [131]. Phase 3 trials investigating acalabrutinib with R-miniCHOP vs. R-miniCHOP in older adults with untreated diffuse large B-cell lymphoma [132] and acalabrutinib with R-CHOP for patients ≤ 65 y with untreated non-germinal center B-cell–like (non-GCB) diffuse large B-cell lymphoma (DLBCL) [133] are ongoing as well. Zanubrutinib also showed efficacy in combination with R-CHOP for the treatment of newly diagnosed double expressor lymphoma in a phase 2 trial [134]. The nuanced efficacy of BTK inhibitors across DLBCL subtypes also suggests that combination therapies, rather than monotherapy, often yield more robust responses.

The SMART START and SMART STOP trials represent pivotal efforts to redefine frontline treatment for diffuse large B-cell lymphoma (DLBCL), particularly for high-risk and non-germinal center B-cell (non-GCB) subtypes. These trials sought to minimize the number of chemoimmunotherapy cycles by incorporating targeted therapy upfront. The SMART START trial was a phase II study that evaluated a targeted therapy combination of rituximab, lenalidomide, and ibrutinib (RLI) administered before and in conjunction with chemotherapy in newly diagnosed non-GCB DLBCL. The ORR after two cycles of RLI was 86.2%, the ORR at EOT was 100%, and the complete response rate at EOT was 94.5% with an acceptable side effect profile. It established the potential for developing biologically driven first-line therapies for DLBCL [135]. Similarly, the SMART STOP trial, a next-generation phase II trial, evaluated the combination of lenalidomide, tafasitamab, rituximab, and acalabrutinib (LTRA) in previously untreated DLBCL. Following four cycles of LTRA, the ORR was 100% and the CRR was 64%, and after an additional two cycles of LTRA-CHOP, the ORR was 100% and the CRR increased to 95%. At the end of all therapy, the CRR is 100%. The most common adverse effect was rash (13% grade 3), and 40% of patients required a dose reduction of lenalidomide. A key aspect of SMART STOP is its adaptive design: patients who achieve a complete response after four cycles of targeted therapy alone may receive only two cycles of R-CHOP instead of the standard six, and a new cohort is exploring the possibility of omitting chemotherapy entirely if deep responses are achieved with the targeted agents alone [136]. These trials have demonstrated the efficacy of targeted therapy as a frontline treatment for DLBCL.

## 7. BCL2 Inhibitors

The CAVALLI study (NCT02055820) investigated the BCL2 inhibitor venetoclax in combination with R-CHOP (rituximab, cyclophosphamide, doxorubicin, vincristine, and prednisone) as first-line treatment for DLBCL [137]. At the end of treatment, CR rates reached 69% across all participants, with 64% in the BCL2 IHC+ group and 66% in patients with double-expressor lymphoma (DEL). Though the 2-year PFS of 78% exceeded historical cohorts of R-CHOP alone, the increased toxicity limited ongoing study of venetoclax in frontline DLBCL [138,139].

The ViPOR trial is investigating venetoclax, ibrutinib, prednisone, obinutuzumab, and lenalidomide in relapsed or refractory DLBCL. It demonstrated an overall response rate of 54%, including a complete response rate of 38% [124]. The highest complete response (CR) rate, approximately 62%, was seen in patients with non-GCB diffuse large B-cell lymphoma. In comparison, CR rates were 53% among those with high-grade B-cell lymphoma featuring BCL2 rearrangement and 25% in other DLBCL subtypes [124]. ViPOR was most effective in non-GCB DLBCL, as expected from its reliance on BCR signaling and BCL2. Its activity in HGBCL-DH-BCL2 may be attributed to BCL2′s role in suppressing MYC-driven apoptosis in these tumors. ViPOR appears to be an active and well-tolerated regimen for heavily pretreated patients, though longer follow-up is needed to determine the durability of responses.

## 8. Tazemetostat and Tulmimetostat

Germinal center DLBCLs depend on the histone methyltransferase EZH2 to stay in a less-differentiated state, and EZH2-activating mutations may be oncogenic drivers in a subset of patients [140]. Inhibition of EZH2 leads to abnormal cell growth, resulting in cell death or differentiation and subsequent tumor regression. Tazemetostat, an oral and highly selective EZH2 inhibitor, has demonstrated antitumor efficacy in DLBCL patients with mutated (mt) or wild-type (wt) EZH2 tumors [140]. The phase II TAZ R-CHOP study (Epi-RCHOP, NCT02889523) evaluated combination therapy of tazemetostat with R-CHOP in patients 60–80 years old with newly diagnosed DLBCL, which showed 75.4% achieved a complete metabolic response (CMR) at the end of treatment with an acceptable safety profile. The estimated 18-month progression-free survival (PFS) was 77.7%, and overall survival (OS) was 88.8% [141]. Tulmimetostat (CPI-0209) is another investigational oral, next-generation, dual EZH2/EZH1 inhibitor. Further studies and clinical trials are ongoing to further evaluate their efficacy in DLBCL.

## 9. Selixenor

XPO1 (exportin 1) is one of the key nucleo-cytoplasmic shuttling proteins involved in the export of proteins from the nucleus to the cytoplasm. It mediates the functional inactivation of multiple tumor suppressor proteins (e.g., p53, p73, IkBκ, FOXO) and facilitates the increased translation of oncoproteins in DLBCL and is often correlated with poor prognosis [142]. Selinexor is an oral selective inhibitor of XPO1-mediated nuclear export that induces the expected nuclear accumulation and activation of tumor suppressor proteins and reduces Bcl2, Bcl-XL, and c-Myc oncoprotein concentrations [142]. The SADAL Phase 2 trial of single-agent oral selinexor showed an overall response rate of 28% and a 12% complete response with a manageable adverse event profile in patients with relapsed or refractory DLBCL who received at least two lines of previous chemoimmunotherapy [142]. The limited efficacy suggests no role for selinexor monotherapy in r/r DLBCL.

## 10. Conclusions

The treatment landscape for diffuse large B-cell lymphoma (DLBCL) has advanced with the introduction of targeted therapies and immunotherapies. For R/R DLBCL, bispecific antibodies, such as epcoritamab and glofitamab, have demonstrated high response rates and manageable safety profiles in R/R DLBCL, including in patients previously treated with CAR-T therapy, and can be more rapidly and widely accessible to patients at community centers. Phase III trials have shown improved outcomes of bispecific antibodies in combination with salvage chemotherapy and/or ADCs in 2L DLBCL, ushering in a new standard for patients ineligible for or unable to access CAR-T or ASCT. Ongoing studies show promise of including bispecifics in frontline DLBCL, but confirmatory phase III trials are ongoing.

Despite the promising efficacy of bispecific antibodies, ongoing challenges remain in their widespread utilization, including multidisciplinary and multi-institutional collaboration for administration and CRS/ICANS management, risk of infections, and financial toxicities.

Antibody–drug conjugates (ADCs) are also approved in frontline and R/R DLBCL. Polatuzumab has improved PFS in high-risk frontline DLBCL when combined with R-CHP and is a new standard of care in many countries/institutions. Chemo-free ADC/bispecific combinations for elderly/unfit patients are also showing early promise in ongoing trials.

Small-molecule inhibitors targeting BTK, BCL2, and EZH2 have also shown efficacy, especially in genetically defined subgroups, suggesting the importance of molecular profiling for treatment optimization. Checkpoint inhibitors have some efficacy but currently have a limited role in heavily pretreated r/r DLBCL with limited duration of response.

These targeted therapies and immunotherapies improve our treatment armamentarium for patients with DLBCL. Randomized trials are underway to determine the long-term efficacy of bispecific antibodies in combination with chemotherapy and/or ADCs in frontline and r/r DLBCL. Ongoing efforts are needed to determine optimal sequencing and combinations of these novel therapies in r/r DLBCL and to better expand access to bispecific antibodies across the globe. Ultimately, these therapies represent exciting new options for patients with DLBCL.

## Figures and Tables

**Table 1:** List of immunotherapies and targeted therapy clinical trials in DLBCL.

Trial Name	Drug(s)	Population	Phase	OR (CR)
Epcoritamab				
EPCORE NHL-1; GCT3013-01 [11]	Subcutaneous Epcoritamab	CD20+ mature B-cell neoplasm (diffuse large B-cell lymphoma (DLBCL) or other aggressive non-Hodgkin lymphomas, including primary mediastinal LBCL, high-grade B-cell lymphoma, or follicular lymphoma grade 3B)	I/II	63 (39)
EPCORE NHL-2 (NCT04663347) [12,13,14,15,16,17]	Epcoritamab plus GemOx [15]	ASCT-ineligible relapsed or refractory (R/R) diffuse large B-cell lymphoma (DLBCL)	IB/II	91 (59)
	Epcoritamab + R-DHAX/C [13]	Transplant-eligible patients (pts) with high-risk re lapsed or refractory (R/R) diffuse large B-cell lymphoma (DLBCL)	IB/II	76 (69)
	Epcoritamab SC + R-mini-CHOP [16]	1L DLBCL who were not considered candidates for full-dose R-CHOP	IB/II	89 (82)
	Epcoritamab + RICE [17]	R/R DLBCL eligible for ASCT	IB/II	87 (65)
EPCORE NHL-5 (NCT05283720)	Epcoritamab Plus Lenalidomide [18]	Relapsed or refractory (R/R) DLBCL	IB/II	67 (51)
	Epcoritamab + pola-R-CHP [19]	Newly diagnosed CD20+ DLBCL	IB/II	100 (97)
EPCORE DLBCL-3 [20,21]	Epcoritamab monotherapy	Elderly pts with 1L CD20+ LBCL who were ineligible for anthracycline-based tx due to age ≥ 80 y or age ≥ 75 y with underlying comorbidities	II	78 (70)
EPCORE DLBCL-1 NCT04628494 [22]	Epcoritamab vs. investigator’s choice of chemotherapy	Relapsed, refractory DLBCL who have failed or are ineligible for high-dose chemotherapy and autologous stem cell transplant (HDT-ASCT)	III	Ongoing
Glotifamab				
STARGLO (NCT0353283) [23]	Glofitamab plus gemcitabine–oxaliplatin (Glofit-GemOx) versus rituximab (R)-GemOx	Transplant-ineligible patients (aged ≥ 18 years) with histologically confirmed relapsed or refractory diffuse large B-cell lymphoma	III	25.5 vs.12.9
NCT03075696 [24,25,26]	Glofitamab + obinutuzumab	Relapsed or refractory DLBCL who had received at least two lines of therapy previously	II	52 (39)
LYSA NCT04703686 [27]	Glofitamab	Refractory or relapsed diffuse large B-cell lymphoma after failing CAR-T cell therapy	II	76 (45)
NCT03533283 [28]	Glofitamab + Polatuzumab Vedotin	Heavily pretreated relapsed/refractory (R/R) large B-cell lymphoma (LBCL), including high-grade B-cell lymphoma (HGBCL)	IB/II	80 (62)
NCT03467373 [29]	Glofitamab + Pola-R-CHP	Previously untreated diffuse large B-cell lymphoma (DLBCL)	IB	100 (76)
SKYGLO NCT06047080 [30]	Glofitamab Plus Polatuzumab Vedotin + Rituximab, Cyclophosphamide, Doxorubicin, and Prednisone (Pola-R-CHP) versus Pola-R-CHP	Previously untreated CD20-positive LBCL	III	Ongoing
NCT04980222 [31]	Glofitamab Plus R-CHOP	Previously untreated (1L) large B-cell lymphoma (LBCL) defined as high risk by circulating tumor DNA (ctDNA) dynamics	II	93 (80)
NCT05364424 [32]	Glofitamab in Combination with Rituximab Plus Ifosfamide, Carboplatin, and Etoposide	Relapsed or refractory diffuse large B-cell lymphoma, eligible for stem cell transplant or chimeric antigen receptor T-cell therapy	IB	78 (68)
NCT04077723 [33]	Englumafusp Alfa + Glofitamab	Relapsed or refractory B-NHL after at least one prior treatment	IB	(aNHL/iNHL) ORR 67.5%/91.7% CR 56.6%/79.2%
COALITION NCT04914741 [34,35]	Glofitamab-R-CHOP or Glofitamab-Polatuzumab Vedotin-R-CHP	Younger patients with high-burden, high-risk large B-cell lymphoma	IB/II	100 (92)
NCT05798156 [34,35]	R-Pola-Glo	Elderly/frail and medically unfit pts with aggressive lymphoma	II	90 (82)
Odronextamab				
ELM-1 study NCT02290951 [36]	Odronextamab	Relapsed/Refractory (R/R) B-cell non-Hodgkin lymphoma after ≥2 lines of therapy—post CAR-T DLBCL cohort	I	48 (31)
ELM-2 study NCT03888105 [37]	Odronextamab	Relapsed/Refractory (R/R) B-cell non-Hodgkin lymphoma after ≥2 lines of therapy—DLBCL cohort	II	52 (31.5)
OLYMPIA-3 (NCT06091865) [38]	Odronextamab plus CHOP vs. rituximab plus CHOP	Previously untreated diffuse large B-cell lymphoma	III	Ongoing
OLYMPIA-4 (EudraCT 2022-502783-21-00) [39]	Odronextamab versus Standard of care	Previously treated aggressive B-NHL	III	Ongoing
Mosunetuzumab				
NCT02500407 [40]	Mosunetuzumab	Relapsed/refractory diffuse large B-cell lymphoma	I/II	42 (24)
NCT03677141 [41]	Mosunetuzumab with CHOP	Previously untreated DLBCL	II	87.5 (85)
NCT03677154 [42]	Mosunetuzumab	Elderly/unfit patients with 1L DLBCL	I/II	67.7 (42)
NCT03671018 [43,44]	Mosunetuzumab + polatuzumab vedotin [43]	Relapsed/refractory aggressive large B-cell lymphoma (LBCL)	Ib/II	59.2 (45.9)
	M (SC administration)-Pola vs. rituximab (R)-Pola [44]		II	OR 78 vs. 50; CR 58 vs. 35
SUNMO (NCT05171647) [45,46]	Mosun + Pola (M-Pola) vs. IV rituximab + gemcitabine and oxaliplatin (R-GemOx)	CD20-positive R/R aNHL, ≥1 prior systemic therapy (if only one prior line of therapy, pts must be ineligible for ASCT)	III	70.3 (51.4)
NCT05672251 [47]	Loncastuximab tesirine (Lonca) + Mosunetuzumab	R/RL DLBCL after ≥2 prior lines of therapy	II	Ongoing
NCT06015880 [48]	Mosunetuzumab, Pola, Lenalidomide	Relapsed/refractory diffuse large B-cell lymphoma	I	Ongoing
Plamotamab				
NCT02924402 [49,50]	Plamotamab IV [49]	Relapsed/refractory (R/R) non-Hodgkin’s lymphoma (NHL)	I	47 (26)
	Plamotamab SC [50]	Heavily pretreated R/R NHL patients who had prior CAR-T cell therapy	I	53 (24)
XmAb13676-03 NCT05328102 [51]	Plamotamab + TAFA + LEN versus TAFA + LEN	DLBCL who have relapsed or are refractory to ≥1 prior line of therapy and are ineligible for or refuse ASCT	II	Ongoing
Blinatumomab				
NCT01741792 [52]	Blinatumomab	Relapsed/refractory DLBCL	II	43 (19)
NCT03340766 [53]	Blinatumomab + Pembrolizumab	Relapsed/refractory DLBCL	IB	30, Terminated due to lack of efficacy
NCT03072771 [54]	Blinatumomab consolidation after auto-SCT	Relapsed diffuse large B-cell lymphoma (DLBCL), who undergo autologous stem cell transplant	I	1 yr—50% CR
NCT03023878 [55]	Blinatumomab following an induction with R- chemotherapy	Adults with newly diagnosed, high-risk DLBCL	II	-
NCT02568553 [56]	Lenalidomide + Blinatumomab	Relapsed or refractory non-Hodgkin’s lymphoma (NHL)	I	Ongoing
Atezolizumab				
HOVON 151 trial NCT03463057 [57]	Atezolizumab	DLBCL patients with an international prognostic index (IPI) score of ≥3 and CMR after R-CHOP	II	2-year: DFS 87.9% OS 96.3%
NCT02220842	Atezolizumab + Obinutuzumab [58]	Relapsed or refractory DLBCL or FL	Ib	17 (4)
	Atezolizumab + Tazemetostat [59]	Relapsed or refractory DLBCL	Ib	16 (7)
NCT02596971 [60]	Atezolizumab + RCHOP	Previously untreated advanced DLBCL	Ib/II	87.5 (77.5)
NCT03321643 [61]	Atezolizumab + rituximab + GemOx (R-GemOx + Atezo)	R/R transformed DLBCL, including Richter transformation	I/II	59 (33)
NCT04082897 MOLTO [62,63]	Atezolizumab + venetoclax + obinutuzumab	Richter transformation diffuse large B-cell lymphoma	II	67.9 (28.6)
NCT03276468 LYSA [64,65]	Atezolizumab + venetoclax + obinutuzumab	Relapsed/refractory B non-Hodgkin lymphoma	II	23.6 (18)
Durvalumab				
NCT03003520 [66]	Durvalumab in combination with R-CHOP or R2-CHOP	Previously untreated, high-risk DLBCL	II	Ongoing; 54% Arm A; 67% Arm B
NCT03685344 [67]	Loncastuximab Tesirine + Durvalumab	R/R DLBCL, MCL, or FL	I/II	Ongoing
Pembrolizumab				
KEYNOTE-170 NCT02576990 [68]	Pembrolizumab	Relapsed/refractory (R/R) primary mediastinal B-cell lymphoma (PMBCL) whose disease progressed after or who were ineligible for autologous stem cell transplantation	II	41 (20)
NCT02332980 [69]	Pembrolizumab Alone or With Idelalisib or Ibrutinib	CLL patients with biopsy-proven Richter transformation to diffuse large B-cell lymphoma	II	23 (7.7)
PORTIA trial NCT03630159 [70]	Tisagenlecleucel + pembrolizumab	Relapsed or refractory DLBCL	Ib	50 (33.3)
KEYNOTE-013 study NCT01953692 [71]	pembrolizumab (cohort 4) or pembrolizumab plus lenalidomide (cohort5)	Relapsed or refractory NHL who were ineligible for or failed hematopoietic cell transplantation (HCT)	Ib	22 (12)/39 (22)
NCT02684617 KEYNOTE-155 [72]	Pembrolizumab + Dinaciclib	Relapsed or refractory DLBCL	Ib	21 (10.5)
NCT03150329 [73]	Pembrolizumab + vorinostat	Relapsed/refractory B-cell NHL—DLBCL cohort	I	55 (45)
KEYNOTE145 NCT02362035 [74]	Acalabrutinib + pembrolizumab	Relapsed/refractory (r/r) diffuse large B-cell lymphoma (DLBCL)	I/II	26 (7)
SPiReL trial NCT03349450 [75]	Pembrolizumab, low-dose cyclophosphamide, +DPX-Survivac	Relapsed/refractory diffuse large B-cell lymphoma (R/R DLBCL)	II	63 (27)
NCT02541565 [76]	Pembrolizumab with R-CHOP	Untreated DLBCL or grade 3b follicular lymphoma		90 (77)
NCT02650999 [77]	Pembrolizumab	Relapsed/refractory B-cell lymphomas after CD19-directed CAR T cells	Ia/II	25
Alexander NCT03287817 [78]	Pembrolizumab + AUTO3, a CAR T targeting CD19/22	r/r DLBCL (NOS) or transformed (tDLBCL)	I	64 (55)
Nivolumab				
CheckMate 139 NCT02038933 [79]	Nivolumab	Relapsed/refractory DLBCL who were ineligible for autologous hematopoietic cell transplantation (auto-HCT) or who had experienced failure with auto-HCT	II	auto-HCT–failed: 10 (3) auto-HCT–ineligible: 3 (0)
NCT03259529 [80]	Nivolumab, bendamustine, gemcitabine + rituximab (BeGeRN)	Relapsed or refractory B-cell non-Hodgkin lymphoma	I/II	45.5 (18)
HOVON-152 NCT03620578 [81]	DA-EPOCH-R Induction and Nivolumab Consolidation Treatment	Newly diagnosed High-grade B-cell lymphoma with MYC and BCL2 and/or BCL6 rearrangements	II	61% CMR
CheckMate 436 NCT02581631 [82]	Nivolumab + Brentuximab Vedotin	Relapsed/refractory primary mediastinal large B-cell lymphoma	II	70 (43)
Polatuzumab Vedotin				
POLARIX NCT03274492 [83,84]	Polatuzumab Vedotin + Rituximab, Cyclophosphamide, Doxorubicin, Prednisone (Pola-R-CHP) vs. R-CHOP	Previously untreated intermediate-risk or high-risk DLBCL	III	2 year PFS: 76.7% (Pola-R-CHP) vs. 70.2% (R-CHOP)
NCT02257567 [85]	Pola + bendamustine + rituximab (Pola + BR) vs. BR alone	Relapsed/refractory diffuse large B-cell lymphoma	Ib/II	BoR:62.5% vs. 25.0% BCR:52.5% vs. 22.5% PFS:9.2 vs. 3.7 mo
NCT04739813 [86]	Polatuzumab, Venetoclax, Ibrutinib, Prednisone, Obinutuzumab, Lenalidomide (ViPOR-P)	Relapsed/refractory diffuse large B-cell lymphoma	I/II	75 (50)
POLARGO NCT04182204 [87]	Pola-R-GemOx vs. R-GemOx	Relapsed/refractory diffuse large B-cell lymphoma	III	52.7 (40.3)
POLAR BEAR NCT04332822 [88]	Pola-R-miniCHP vs. R-miniCHOP	Newly diagnosed DLBCL, >80 years, or 75–80 years and frail	III	Ongoing
NCT04231877 PERCH [89]	Pola, etoposide, cyclophosphamide, doxorubicin, rituximab (Pola-DA-EPCH-R)	Aggressive large B-cell lymphomas	I	93 (71)
NCT04594798 [90]	Pola, Rituximab and Dose Attenuated CHP	Newly diagnosed DLBCL patients 75 years and older	II	Ongoing
NCT04665765 [91]	Pola, rituximab, ifosfamide, carboplatin, etoposide (PolaR-ICE)	First salvage therapy for relapsed or refractory (R/R) diffuse large B-cell lymphoma (DLBCL)	II	Ongoing
NCT04833114 [92]	Pola, rituximab, ifosfamide, carboplatin, etoposide (Pola-R-ICE) vs. rituximab, ifosfamide, carboplatin and etoposide (R-ICE)	Salvage therapy in patients with primary refractory or relapsed diffuse large B-cell lymphoma (DLBCL)	III	Ongoing
NCT04479267 [93]	Pola, Rituximab, Cyclophosphamide, Doxorubicin, Prednisone	Patients aged > 18 years with previously untreated double- or triple-hit lymphoma	II	Ongoing
NCT06176729 [94]	Pola, Rituximab Lenalidomide (Pola-R2)	Newly diagnosed DLBCL patients aged over 70 years old and unfit or frail	II	EOT CRR 100%
NCT06743945 [95]	POLA-R-CHP	Patients with transformed DLBCL	II	Ongoing
NCT06530511 [96]	Polatuzumab, Rituximab, Orelabrutinib (PRO)	Elderly patients with frail, treatment-naïve, non-germinal center subtype diffuse large B-cell lymphoma	II	Ongoing
NCT06468943 [97]	Pola, Zanubrutinib, R-CHP	Newly diagnosed untreated non-GCB DLBCL patients with extranodal involvement	II	Ongoing
NCT06015880 [48]	Mosunetuzumab, Pola, Lenalidomide	Relapsed/refractory diffuse large B-cell lymphoma	I	Ongoing
NCT07001540 [98]	Pola-R-GemOx	Salvage therapy for relapsed/refractory diffuse large B-cell lymphoma (DLBCL) patients ineligible for autologous transplantation	II	Ongoing
NCT06664411 [99]	Pola, Zanubrutinib, Rituximab, Lenalidomide Prednisone Pola-ZR2P	Previously untreated DLBCL	II	Ongoing
NCT02611323 [100]	Pola, Venetoclax, Rituximab Pola-Ven-R	Relapsed/refractory (R/R) diffuse large B-cell lymphoma (DLBCL)	Ib/II	65 (31)
Loncastuximab tesirine				
LOTIS 2 NCT03589469 [101]	Loncastuximab	Relapsed/refractory diffuse large B-cell lymphoma	II	48 (25)
LOTIS-3 NCT03684694 [102]	Loncastuximab + ibrutinib	Relapsed/refractory diffuse large B-cell lymphoma	I/II	57 (34)
LOTIS-5 NCT04384484 [103]	Lonca-R vs. R + gemcitabine + oxaliplatin (R-GemOx)	Relapsed/refractory diffuse large B-cell lymphoma	III	Ongoing Prelim: 80 (50)
LOTIS-7 NCT04970901 [104]	Lonca + Pola (arm C), glofitamab (arm E), or mosunetuzumab (arm F)	Relapsed/refractory diffuse large B-cell lymphoma	Ib	Lonca + Glofit 95 (90.9)
LORELY NCT06918912 [105]	Loncastuximab	Relapsed/refractory diffuse large B-cell lymphoma (DLBCL) or high-grade B-cell lymphoma (HGBCL) following CAR-T therapy failure	II	Ongoing
Brentuximab vedotin				
NCT01421667 [106]	Brentuximab	Relapsed/refractory diffuse large B-cell lymphoma	II	44 (17)
ECHELON-3 NCT04404283 [107]	Brentuximab vedotin, lenalidomide, rituximab vs. R2	Relapsed/refractory diffuse large B-cell lymphoma	III	70.9 (45.6)
NCT02734771 [108]	Brentuximab Vedotin, Rituximab, Dose Attenuated CHP	Elderly patients with newly diagnosed DLBCL	II	Ongoing
NCT01994850 [109]	Brentuximab vedotin (BV) rituximab, cyclophosphamide, doxorubicin, prednisone (R-CHP)	CD30-positive (+) B-cell lymphomas	I/II	100 (86)
Zilovertamab Vedotin				
waveLINE-011 NCT06890884 [110]	Zilovertamab Vedotin +R-CHP vs. Pola + R-CHP	Treatment-naïve DLBCL, GCB subtype	II	Ongoing
waveLINE-007 NCT05406401 [111]	Zilovertamab vedotin + R-CHP	Previously untreated DLBCL	II	100 (100)
waveLINE-010 NCT06717347 [112]	Zilovertamab vedotin + R-CHP vs. R-CHOP	Previously untreated DLBCL	III	Ongoing
waveLINE-003 NCT05139017 [113]	Zilovertamab vedotin, rituximab, gemcitabine-oxaliplatin (R-GemOx)	Relapsed/refractory DLBCL	II/III	56
waveLINE-004 NCT05144841 [114]	Zilovertamab vedotin	Relapsed/refractory DLBCL	II	29 (13)
Naratuximab Emtansine				
NCT02564744 [115]	Naratuximab Emtansine, rituximab	Relapsed and/or refractory (R/R) B-NHL	II	44 (31)

Abbreviations: R/R = relapsed/refractory; DLBCL = diffuse large B-cell lymphoma; ASCT = autologous stem cell transplant; CR = complete response; ORR = overall response rate; PFS = progression-free survival; OS = overall survival; NE = not estimable.
